# The Effects of Powered Exoskeleton Gait Training on Cardiovascular Function and Gait Performance: A Systematic Review

**DOI:** 10.3390/s21093207

**Published:** 2021-05-05

**Authors:** Damien Duddy, Rónán Doherty, James Connolly, Stephen McNally, Johnny Loughrey, Maria Faulkner

**Affiliations:** 1 Sports Lab North West, Letterkenny Institute of Technology, Port Road, Letterkenny, F92 FC93 Donegal, Ireland; Ronan.Doherty@lyit.ie (R.D.); Maria.Faulkner@lyit.ie (M.F.); 2 Department of Computing, Letterkenny Institute of Technology, Port Road, Letterkenny, F92 FC93 Donegal, Ireland; James.Connolly@lyit.ie; 3 No Barriers Foundation, Letterkenny, F92 TW27 Donegal, Ireland; stephen@nobarriers.ie (S.M.); johnny@jtphysio.com (J.L.)

**Keywords:** energy expenditure, oxygen consumption, heart rate, exoskeleton, spinal cord injury

## Abstract

Patients with neurological impairments often experience physical deconditioning, resulting in reduced fitness and health. Powered exoskeleton training may be a successful method to combat physical deconditioning and its comorbidities, providing patients with a valuable and novel experience. This systematic review aimed to conduct a search of relevant literature, to examine the effects of powered exoskeleton training on cardiovascular function and gait performance. Two electronic database searches were performed (2 April 2020 to 12 February 2021) and manual reference list searches of relevant manuscripts were completed. Studies meeting the inclusion criteria were systematically reviewed in accordance with Preferred Reporting Items for Systematic Reviews and Meta-Analyses (PRISMA) guidelines. *n* = 63 relevant titles were highlighed; two further titles were identified through manual reference list searches. Following analysis *n* = 23 studies were included. Data extraction details included; sample size, age, gender, injury, the exoskeleton used, intervention duration, weekly sessions, total sessions, session duration and outcome measures. Results indicated that exoskeleton gait training elevated energy expenditure greater than wheelchair propulsion and improved gait function. Patients exercised at a moderate-intensity. Powered exoskeletons may increase energy expenditure to a similar level as non-exoskeleton walking, which may improve cardiovascular function more effectively than wheelchair propulsion alone.

## 1. Introduction

Walking is a primary component of human movement, which requires activation of the lower limbs in order to step and support body mass [1]. Walking can increase physical workload, which places a greater demand on the cardiorespiratory system to deliver oxygen to the working muscles [2]. Walking is the most popular recreational activity in Ireland, as 18% of able-bodied adults (18–64 years old) meet the physical activity guidelines of at least 30 min of moderate intensity activity per day, five days per week (150 min in total), or alternatively a total of 75 min of vigorous intensity activity per week [3] by walking [4]. On average, recreational walkers complete greater than four walks per week, 80% of which last at least 30 min and 93% are completed at an average speed or faster [4]. According to Finley and Cody, the average walking speed for healthy adults is approximately 1.3 m·s^−1^ (males: 1.37 m·s^−1^, females: 1.23 m·s^−1^), which equates to 4.68 km·h^−1^ [5]. For able-bodied individuals, simultaneously increasing the stimulation of the cardiorespiratory system and the activation of the lower limbs can potentially increase metabolic rate by up to eight times higher than rest (one MET) [6]; one MET is approximately equal to an oxygen consumption rate of 3.5 mL·kg^−1^·min^−1^ [6]. For example, walking at speeds of 5.6 and 8.0 km·h^−1^ can produce metabolic rates of 3.8 and 8 METs, respectively [7]. As a result, walking in accordance with physical activity guidelines at a moderate (3–5.9 METs) or vigorous (≥6 METs) intensity [8] can be an effective way of increasing energy expenditure, improving cardiorespiratory fitness and enhancing health [6]. This in turn may help reduce the risk of developing cardiovascular and metabolic diseases [9] such as, heart disease, obesity, lipid disorders, metabolic syndrome, and diabetes [10]. Anton et al. determined that walking at a vigorous intensity (65–75% HRres) for a shorter duration (≥60 min per week) yielded greater improvements in cardiorespiratory fitness than walking at a moderate intensity (45–55% HRres) for a longer duration (≥150 min per week) [11].

Due to a reduction in gait and mobility, individuals with neurological impairments such as multiple sclerosis (MS) [12], stroke [13] and spinal cord injury (SCI) [10] tend to develop a predominantly sedentary lifestyle, involving long durations of sitting [10]. As well as reduced motor function, limited accessibility is a major contributor to a sedentary lifestyle among patients with neurological impairments, with few opportunities to participate in physical activity [10], leading to a reduction in quality of life (QoL) [12]. Therefore, the main option for mobility for individuals with paraplegia is a wheelchair [14]. As a result of a predominantly sedentary lifestyle, individuals with paraplegia often experience some physical deconditioning, especially as they age. Physical deconditioning often reduces cardiovascular fitness and may lead to a number of chronic secondary health problems [15] such as diabetes mellitus and obesity [16]. Furthermore, physical deconditioning can rapidly decrease the QoL of SCI patients by reducing cardiorespiratory and muscular function which may impair mobility, resulting in the patients being completely physically dependent, relying on help for mobility issues and social roles [17]. The loss of physical fitness and mobility negatively impacts the patients’ ability to carry out daily tasks, resulting in poor social interaction [17]. This highlights the importance of being physically active in order to improve health, fitness and overall QoL [15]. It is suggested that the mortality rate among patients with chronic SCI is elevated by up to 47%. Risk factors influencing mortality include heart disease, diabetes and reduced pulmonary function [16]. The standard mortality rates for motor complete SCI and motor incomplete SCI were 1.41 (CI = 0.77–2.37) with 14/9.92 observed/expected deaths, and 1.23 (CI = 0.61–2.21) with 11/8.92 observed/expected deaths, respectively [16]. The development of cardiovascular disease and the occurrence of an ischemic stroke is more prevalent among SCI patients in comparison to their able-bodied counterparts [18]. In order to diminish secondary health problems and enhance cardiovascular fitness, mainly by improving aerobic capacity [19], patients should comply with the updated physical activity guidelines for health published by the World Health Organisation (WHO) [20]. Adults living with disability (MS and SCI) should complete at least 150 to 300 min of moderate intensity aerobic activity or at least 75 to 150 min of vigorous intensity aerobic activity, or an equivalent combination of both per week [20]. It is also recommended that adults with disabilities undertake muscle strengthening activities at a moderate intensity or greater, involving all the major muscle groups at least twice per week [20]. However, Ginis et al. suggested that SCI patients should complete 20 min of moderate to vigorous intensity activity twice per week [15]. For individuals with paraplegia, the typical methods used to achieve prolonged bouts of moderate-intensity exercise would be using an arm crank or wheelchair ergometer, whereby the majority of muscle mass recruited to perform these exercises are located in the upper extremities [21]. 

**Figure 1:**
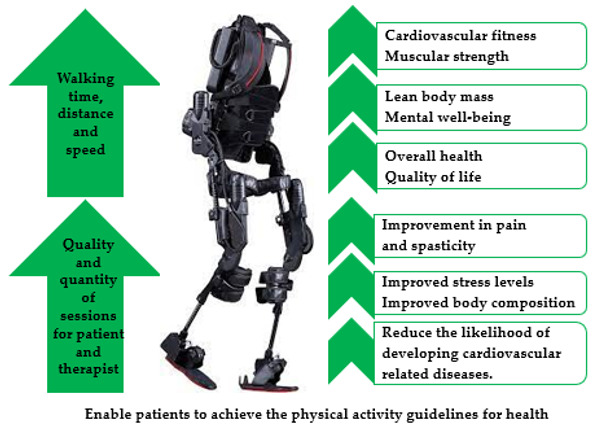
Benefits of powered exoskeleton gait training.

However, robotic exoskeletons enable wheelchair users with little or no walking ability to walk over ground, with maximal external support [21]. Prolonged bouts of robotic exoskeleton assisted walking have the potential to produce a moderate-intensity level of exercise, and may provide adequate stimulus to enhance cardiovascular fitness within an SCI population [22]. Existing evidence has indicated that robotic exoskeleton training may contribute to the reduction of secondary health complications among SCI patients, by increasing bone mineral density and lean body mass, as well as reducing spasticity, improving bowel function, and enhancing gait function [23,24]. Furthermore, previous research has also highlighted that robotic exoskeleton walking reduced physical pain and improved QoL among individuals with paraplegia [25] (Figure 1). According to Portaro et al. powered exoskeleton training can improve both musculoskeletal and neuromuscular performance and may also contribute to neuroplasticity [26]. Therefore, incorporating a robotic exoskeleton into a rehabilitation programme may enhance physical fitness to a greater level and minimise secondary health problems more effectively than a wheelchair ergometer or an arm crank [21]. The purpose of this systematic review was to evaluate and review existing literature that examined energy expenditure and gait performance associated with powered exoskeleton assisted walking.

## 2. Materials and Methods

### 2.1. Study Design

This systematic review was designed to include and evaluate research on the effects of powered exoskeleton training on cardiovascular function and gait performance.

### 2.2. Search Strategy

PRISMA guidelines were followed to conduct a search of current literature surrounding the topic area [27]. Specific search terms were developed to highlight the most relevant research. The strategy consisted of searching multiple databases with the specific search terms, e.g., “energy expenditure” of “powered exoskeleton” ambulation, “cardiorespiratory demands” of “powered exoskeleton” walking, “metabolic demands” of “powered exoskeleton” walking, Ekso GT energy cost, “powered exoskeleton” “oxygen demand” paraplegic, powered exoskeleton “physiological cost” paraplegic, and powered exoskeleton “energy expenditure” paraplegic (Appendix A). The search terms included the Ekso GT as the authors are currently conducting research surrounding the device. Databases searched included Google Scholar and ScienceDirect, the search was conducted between 2 April 2020 and 12 February 2021. The terms were searched on both databases, relevant titles were highlighted and abstracts assessed. If the title and abstract met the inclusion criteria the full study was examined. If the full manuscript was relevant and met the inclusion criteria, the study was included in this systematic review. 

### 2.3. Study Selection

**Figure 2:**
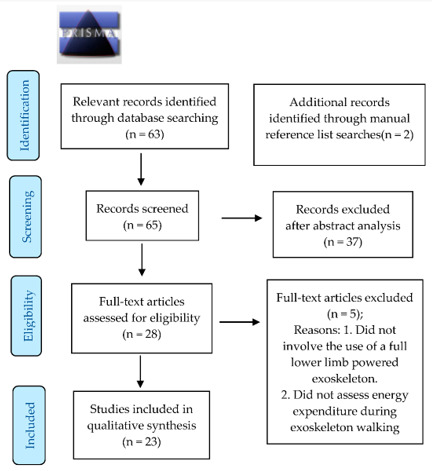
Prisma Flow Chart.

To be eligible for inclusion, studies must have involved the use of a full lower limb powered exoskeleton and examined an element of energy expenditure or cardiovascular function associated with exoskeleton assisted walking. Studies were excluded if they did not involve the use of a full lower limb powered exoskeleton and did not assess an element of energy expenditure during exoskeleton assisted walking. The search resulted in *n* = 63 relevant titles with two further relevant titles identified through references list searches. Following abstract analysis (*n* = 65), *n* = 37 studies were excluded as they did not meet the inclusion criteria. *n* = 28 entire manuscripts were examined and a total of *n* = 23 studies were included (Figure 2).

### 2.4. Data Extraction

The data extracted from the selected studies consisted of participant details such as, sample size, age, gender and injury type (Table 1). The exoskeleton device name and intervention protocols such as, the duration of the intervention, number of sessions per week, total number of sessions and length of sessions were documented (Table 1). Outcome measures and findings were also recorded (Table 1).

### 2.5. Outcome Measures 

All included studies (*n* = 23) calculated various objective and subjective measurements of energy expenditure associated with powered exoskeleton training. Volume of oxygen ($\dot{V}$O_2_); which was reported in relative and absolute terms including, millilitres per kilogram of body mass per minute (mL·kg^−1^·min^−1^), millilitres per kilogram of body mass per kilometre (mL·kg^−1^·km^−1^), litres per minute (L·min^−1^) or millilitres (mL). Heart rate (HR; bpm), was measured. Rate of perceived exertion (RPE) was self-reported using a Borg scale and the metabolic equivalent of task (METs) was measured to assess exercise intensity. Physiological cost index (PCI; bpm), respiratory exchange ratio (RER), the number of calories expended per minute (kcal·min^−1^) and blood pressure (BP; mmHg) were also recorded (Table 1). 

Some included studies (*n* = 21) also measured an element of gait performance. The authors assessed variables such as; 25-foot walk test (25FWT) which recorded time over a 25-foot walk, six-minute walk test (6 MWT) that recorded total distance covered over six minutes of walking. Time up and go test (TUG), which measured the time needed to stand up, balance, walk three metres and back, and sit down. Ten-metre walk test (10 MWT) which recorded the time over a ten-metre walk distance. Two-minute walk test (2 MWT) which measured distance covered over a two-minute walking duration. The 30-min walk test (30 MWT) which recorded distance over a walking time of 30 min. Total steps, overall distance and walking speed were also recorded in some studies (Table 1).

## 3. Results

### 3.1. Study Characteristics

The design of the selected studies included case series/reports (*n* = 7) [34,35,36,41,42,44,47], pilot studies (*n* = 1) [28], prospective open label (*n* = 1) [39], self-controlled feasibility (*n* = 1) [31], longitudinal cohort (*n* = 1) [40], prospective cohort (*n* = 1) [38], randomised crossover trial (*n* = 2) [37,43], open label prospective quasi-experimental (*n* = 1) [30], prospective single group observational (*n* = 1) [14], experimental (*n* = 5) [22,29,32,33,45], cross sectional (*n* = 1) [21] and comparative (*n* = 1) [46] studies. The studies were conducted in the USA (*n* = 13) [14,22,28,32,34,35,37,39,40,41,42,44,45], UK (*n* = 2) [29,31], Canada (*n* = 2) [21,38], Korea (*n* = 2) [36,43], Italy (*n* = 2) [33,47] and Australia (*n* = 1) [46]; one study was conducted in Denmark, Germany, the Netherlands, Norway, Spain and Sweden [30], collectively involving 236 participants (*n* = 236). Across the included studies (*n* = 23), the participants’ ranged from 18 to 67 years of age. The gender of participants was not disclosed by one study, the remaining studies (*n* = 22) were comprised of 151 males and 65 females (Table 1). The participants from *n* = 19 studies [14,21,22,29,30,31,33,34,35,36,38,39,40,41,42,43,44,45,47] were SCI patients, one study involved individuals with MS [28], another study consisted of stroke patients [37] and one study involved able-bodied participants [32]. One study involved patients with MS and stroke as well as healthy individuals [46]. All selected studies (*n* = 23) used a powered exoskeleton (Table 1).

### 3.2. Protocols

The powered exoskeleton devices used throughout the selected studies included the EKSO (*n* = 6) [28,30,35,40,41,44], Ekso GT (*n* = 5) [21,30,33,42,45], ReWalk (*n* = 6) [14,31,38,39,43,46], Indego (*n* = 2) [22,34], Powered Gait Orthosis (PGO) (*n* = 1) [29], Angelegs (*n* = 1) [36], Hybrid Neuro-Prosthesis Exoskeleton (*n* = 1) [32], SMA Exoskeleton (*n* = 1) [37] and REX Bionics (*n* = 1) [46]. Both the EKSO and Ekso GT were included in the intervention of one study [30]. The intervention length applied in the selected studies ranged from one to 24 weeks, accumulating between one and 60 total sessions using a powered exoskeleton. Session duration ranged from 30 min to two hours, with participants completing between one and five sessions per week (Table 1).

### 3.3. Cardiovascular Function

#### 3.3.1. $\dot{V}$O_2_

In total, *n* = 16 studies recorded $\dot{V}$O_2_ associated with powered exoskeleton assisted walking (Table 2). Oxygen consumption was examined during a seated, standing and walking element in five studies. Across four of the studies, $\dot{V}$O_2_ ranged from 2.58 ± 0.67 to 4.3 ± 1.12 mL·kg^−1^·min^−1^ when seated, 3.02 ± 0.48 to 4.7 ± 0.58 mL·kg^−1^·min^−1^ when standing and 7.2 ± 1.9 to 11.2 ± 1.7 mL·kg^−1^·min^−1^ when walking with powered exoskeleton assistance within an SCI population [14,21,33,45]. Maher et al. also assessed a seated (4.2 ± 0.44 mL·kg^−1^·min^−1^), standing (4.8 ± 0.45 mL·kg^−1^·min^−1^) and walking (11.3 ± 1.30 mL·kg^−1^·min^−1^) element among an able-bodied control group [45]. A standing (2.67 ± 0.57 mL·kg^−1^·min^−1^) and walking (3.91 ± 0.93 mL·kg^−1^·min^−1^) element was also examined by Corbianco et al. using a lokomat, which produced significantly lower (*p* < 0.001) mean $\dot{V}$O_2_ recordings when compared to the Ekso GT [33] (Table 2). Gorgey et al. reported $\dot{V}$O_2_ when resting (0.27 L·min^−1^) when standing (0.4 L·min^−1^), and when walking (0.55 L·min^−1^) with powered exoskeleton assistance after a 12-week walking intervention [35]. Oxygen consumption was recorded at pre-and-post-intervention in four studies, across two of the studies a slight reduction in range was present from pre (8.40–38.90 mL·kg^−1^·min^−1^) to post (9.38–32.9 mL·kg^−1^·min^−1^) intervention [39,41]. Jang et al. also indicated a reduction in $\dot{V}$O_2_ results from pre (1208.1 mL) to mid (1077.9 mL) and from mid to post (901.3 mL) intervention [36], whereas Postol et al. highlighted an increase in $\dot{V}$O_2_ from pre (3.7 ± 0.7 mL·kg^−1^·min^−1^) to post (5.3 ± 1.9 mL·kg^−1^·min^−1^) intervention among patients with neurological impairments during a five-minute walking bout [46]; which was slightly lower than healthy individuals walking with exoskeleton assistance (7.0 ± 2.3 mL·kg^−1^·min^−1^) [46]. Afzal et al. calculated N$\dot{V}$O_2_-peak during the 6 MWT and 25FWT at both a self-selected (SS) speed and fast speed (FS) at pre-and-post-intervention [28]; N$\dot{V}$O_2_-peak is described as the difference between $\dot{V}$O_2_ peak when walking and standing. Results demonstrated a slight increase in N$\dot{V}$O_2_-peak from pre-intervention (5.76 ± 1.3 N$\dot{V}$O_2_) to post-intervention (5.91 ± 1.2 N$\dot{V}$O_2_) during the 6 MWT. N$\dot{V}$O_2_-peak results during the 25FWT reduced slightly from pre-intervention at a SS speed (2.83 ± 1.1 N$\dot{V}$O_2_) and FS (3.60 ± 1.3 N$\dot{V}$O_2_) to post-intervention at a SS speed (2.01 ± 1.0 N$\dot{V}$O_2_) and FS (3.31 ± 1.9 N$\dot{V}$O_2_) [28]. When walking powered exoskeleton assistance, a mean $\dot{V}$O_2_ improvement of 34.92 ± 14.84 mL·kg-^1^·km^−1^ during the 6 MWT and a peak $\dot{V}$O_2_ improvement of 0.08 ± 0.04 mL·kg^−1^·km^−1^ during the graded treadmill endurance test were made, in comparison to unpowered assistance [37]. Oxygen consumption during powered exoskeleton assisted walking was recorded in five studies; which consisted of SCI patients and able-bodied individuals. The mean results across the five studies collectively ranged from 9.0 ± 2.1 to 22.5 ± 3.4 mL·kg^−1^·min^−1^ [22,32,42,43,47]. Chang et al. also examined the $\dot{V}$O_2_ of non-exoskeleton assisted walking among able-bodied participants which was reported as 11.7 ± 2.0 mL·kg-^1^·min^−1^ [32], which was similar to unpowererd knee, ankle and foot ortosis (KAFO) (11.8 ± 1.8 mL·kg^−1^·min^−1^) among SCI patients [43].

#### 3.3.2. HR

HR data was collected in a total of *n* = 12 studies, using a range of polar HR monitors (Table 3). HR was examined during a seated, standing and walking period in four studies, the range across the four studies increased from 58 to 95 bpm when seated compared to 61 to 108 bpm when standing, and HR during the walking element ranged from 87 to 146 bpm [14,21,33,47]. Bach Baunsgaard et al. showed an increase in HR (15–21%) from the seated to the walking element at three-time points [30]. A further three studies examined HR before and after one training session, across the three studies the collective results ranged from 52 to 115 bpm pre-training and from 53 to 135 bpm post-training [31,40,44]. Kressler et al. recorded the mean peak HR which increased from pre (166.6 ± 24.0 bpm) to post (172.6 ± 5.13 bpm) intervention [41]. The remaining authors recorded HR during powered exoskeleton assisted ambulation, which collectively ranged from 57 to 177 bpm across four studies [22,32,40,43], and was considerably higher than able-bodied individuals walking without exoskeleton assistance (80–113 bpm) [32], and was significantly higher (*p* < 0.001) than walking with the lokomat (84 ± 9 bpm) [33]. According to Kwon et al. walking with KAFO produced a much higher HR during the 6 MWT (mean; 129.1 ± 19.1 bpm, peak; 145.3 ± 19.3 bpm) and 30 MWT (mean; 143.9 ± 14.6 bpm, peak; 160.5 ± 17.5 bpm) when compared to a powered exoskeleton [43] (Table 3).

#### 3.3.3. RPE

As illustrated in Table 3, five studies recorded RPE during exoskeleton assisted walking. A total of three studies used a Borg scale which ranged from 6 to 20; across the three studies the combined results ranged from 7 to 13, which is scaled as extremely light to somewhat hard [14,30,40]. Escalona et al. recorded RPE using a Borg scale which ranged from 1 to 10, the mean score during exoskeleton ambulation was 3.2, which is equal to moderate intensity activity [21]. Knezevic et al. assessed the mean RPE over 60 sessions and found a significant reduction (*p* = 0.001) from somewhat hard (13 ± 5.95) to very, very light (7 ± 3.52) [39].

#### 3.3.4. METS

A total of six studies calculated METs during powered exoskeleton assisted walking. Chang et al. examined the mean METs of walking with (6.5 ± 1.0 METs) and without (3.4 ± 0.6 METs) exoskeleton assistance among able-bodied individuals [32]. Evans et al. recorded METs over two six-minute bouts of exoskeleton assisted walking, with a five-minute rest period between each walking bout [22]. The results increased from walk one (3.5 ± 0.3 METs) to walk two (4.26 ± 0.51 METs) [22]. Jang et al. reported a reduction in peak METs from pre (4.6 METs) to mid (4.1 METs) and a further reduction from mid to post (3.4 METs) intervention [36]. Kozlowksi et al. recorded the mean METs over a 24-week intervention (2.3 METs), which ranged from 0.6 to 3.9 METs [40]. Corbianco et al. stated that METs during powered exoskeleton gait training (3.2 ± 1.01 METs) were significantly greater (*p* < 0.001) than walking with the lokomat (1.58 ± 0.44 METs) within an SCI population [33], whereas Kwon et al. highlighted that METs when walking with a powered exoskeleton (6 MWT: 2.6 ± 0.6 METs, 30 MWT: 2.5 ± 0.5 METs) were less than when walking with unpowered assistance (6 MWT: 3.4 ± 0.5 METs, 30 MWT: 3.6 ± 0.7 METs) [43].

#### 3.3.5. PCI

PCI during exoskeleton assisted walking was calculated in four studies. The combined PCI findings across the four studies ranged from 0.92 ± 0.38 to 1.60 ± 0.84 bpm during powered exoskeleton assisted walking among SCI and able-bodied participants [29,32,34,38]. Chang et al. reported that PCI during powered exoskeleton assisted walking (1.1 bpm) which ranged from 0.74 to 1.28 bpm, was at least 4.3-fold greater than non-exoskeleton walking among able-bodied individuals (0.2 bpm) which ranged from 0.15 to 0.28 bpm [32]. The results collected by Khan et al. indicated that PCI during powered exoskeleton assisted walking (1.60 ± 0.84 bpm) was at least three times greater than PCI during wheelchair propulsion (0.49 ± 0.09 bpm) within an SCI population, which was similar to PCI during over ground walking among uninjured patients (0.52 ± 0.14 bpm) [38]. Arazpour et al. highlighted that PCI during powered gait orthosis (0.92 ± 0.25 bpm) was slightly less than walking with unpowered assistance such as; the hip knee ankle foot orthosis (HKAFO) (1.97 ± 0.17 bpm) and isocentric reciprocating gait orthosis (IRGO) (1.93 ± 0.40 BPM) [29]. Similarly, Farris et al. stated that a powered exoskeleton produced a slightly lower PCI during TUG (4.73 ± 1.95 bpm), 10 MWT (0.52 ± 0.18 bpm) and 6 MWT (0.92 ± 0.38 bpm), when compared to HKAFOs during TUG (7.7 ± 0.79 bpm), 10 MWT (2.69 ± 0.83 bpm) and 6 MWT (2.97 ± 0.93 bpm) [34]. Kwon et al. also highlighted that a lower PCI was produced during powered exoskeleton walking (6 MWT: 4.7 ± 1.4 bpm, 30 MWT: 5.7 ± 1.8 bpm) in comparison to KAFOs (6 MWT: 5.6 ± 3.2 bpm, 30 MWT: 8.3 ± 3.3 bpm) [43].

#### 3.3.6. RER

RER was assessed during powered exoskeleton assisted walking in a total of four studies. During a seated, standing and walking element, mean RER ranged from 0.8 to 1.0 [21]. Carbohydrate (CHO) utilisation and fat oxidation rates were examined by Kressler et al. and were expressed as grams per minute (g·min^−1^), CHO utilisation was assessed when seated (0.10–0.37 g·min^−1^), standing (0.08–0.30 g·min^−1^) and when walking (0.16–0.85 g·min^−1^) with maximal exoskeleton assistance [42]. Fat oxidation was considerably lower when seated (0–0.06 g·min^−1^), when standing (0.01–0.14 g·min-^1^) and when walking (0–0.11 g·min^−1^) with maximal support [42]. Kressler et al. recorded CHO and fat oxidation percentages during exoskeleton assisted walking at baseline (15–74% CHO, 36–85% Fat), after nine sessions (23–46% CHO, 54–77% Fat) and after 18 sessions (19–54% CHO, 46–81% Fat) [41]. Maher et al. assessed CHO and Fat oxidation percentages during a seated (40% CHO, 60% fat), standing (30% CHO, 70% fat) and exoskeleton walking (45% CHO, 55% fat) element among SCI patients [45]; and among an able-bodied control group during a seated (45% CHO, 55% fat), standing (35% CHO, 65% fat) and walking (40% CHO, 60% fat) element [45].

#### 3.3.7. Energy Expenditure

Gorgey et al. and Maher et al. examined energy expenditure (kcal·min^−1^) during a seated, standing and walking period within an SCI population [35,45]. Post-intervention Gorgey et al. displayed an increase in energy expenditure from seated (1.3 kcal·min^−1^) to standing (2.05 kcal·min^−1^) and a further increase when walking (2.7 kcal·min^−1^), within a single patient [35]. A similar trend was presented by Maher et al. when seated (1.38 ± 0.30 kcal·min^−1^), standing (1.52 ± 0.09 kcal·min^−1^) and walking with exoskeleton assistance (2.81 ± 0.35 kcal·min^−1^), within SCI patients [45]. Maher et al. also assessed energy expenditure among an able-bodied control group during a seated (1.65 ± 0.30 kcal·min^−1^), standing (1.87 ± 0.37 kcal·min^−1^) and walking period (4.46 ± 0.98 kcal·min^−1^) [45]. Kressler et al. examined energy expenditure during powered exoskeleton ambulation which ranged from 1.72 to 7.17 kcal·min^−1^ [42]. Energy expenditure during exoskeleton walking was also assessed at baseline (1.53–2.26 kcal·min^−1^), after nine sessions (1.38–2.75 kcal·min^−1^) and post-intervention after 18 sessions (1.39–2.56 kcal·min^−1^) [41]. Kwon et al. examined energy expenditure during the 6 MWT (3.0 ± 0.8 kcal·min^−1^) and 30 MWT (2.9 ± 0.7 kcal·min^−1^) using a powered exoskeleton, which was slightly less than using unpowered assistance during the 6 MWT (3.9 ± 0.6 kcal·min^−1^) and 30 MWT (4.0 ± 1.0 kcal·min^−1^) among SCI patients [43].

#### 3.3.8. BP

Lester and Gorgey, recorded BP pre-and-post two exoskeleton training sessions, BP changed from 111/77 mmHg to 117/74 mmHg after session one, and increased from 88/58 mmHg to 122/82 mmHg after session two [44]. Gorgey et al. also reported a slight improvement in BP from resting pre-training (85/55 mmHg) to post-training (86/58 mmHg) [35].

### 3.4. Gait Performance

#### 3.4.1. 6 MWT

As well as assessing energy expenditure, eight studies also employed the 6 MWT as a measurement of gait performance (Table 4). The 6 MWT was recorded at pre-intervention in two studies, the mean distances recorded in both studies were 121.2 ± 64.7 m and 51.1 ± 51 m, respectively [28,39]. The 6 MWT was examined at mid intervention in two studies; the mean results were 117.6 m and 71.9 ± 45.1 m, respectively, within an SCI population [31,39]. A total of four studies recorded the 6 MWT at post-intervention, the mean results collectively ranged from 98.9 ± 42.3 to 146.3 ± 35.3 m [28,31,38,39]. Benson et al. also conducted the 6 MWT without powered exoskeleton assistance pre (11–135 m) and post-intervention (19–135 m) among SCI patients [31]. The 6 MWT was also recorded by Knezevic et al. without a metabolic analyser at pre (47.2 ± 45.9 m), mid (83.5 ± 51.5 m) and post (117.9 ± 38.9 m) intervention [39]. Evans et al. recorded two 6 MWT with powered exoskeleton assistance, with a five-minute recovery period between both walks [22]. The mean distance increased from walk one (67.4 ± 3.8 m; range 62.83–72.05 m) to walk two (95.9 ± 18.6 m; range 81.30–128.46 m) [22]. Farris et al. examined 6 MWT distance with powered (64 ± 4.5 m) and unpowered (37 ± 1.9 m) exoskeleton assistance [34]; similarly, Jayaraman et al. noted a 32.3 ± 15.5 m improvement in 6 MWT distance with powered versus unpowered exoskeleton assistance [37]. Conversely, Kwon et al. indicated that greater distance was covered during the 6 MWT using an unpowered KAFO (50.9 ± 25.5 m) in comparison to a powered exoskeleton (39.1 ± 5.4 m), whereby patients walked faster with the KAFO (9.6 ± 4.2 m·min^−1^, 25.2 ± 6.3 steps·min^−1^) when compared to the exoskeleton (6.0 ± 2.4 m·min^−1^, 20.9 ± 7.1 steps·min^−1^) [43].

#### 3.4.2. 10 MWT

The 10 MWT was employed in seven studies (Table 4). Out of the seven studies, four studies recorded the time required to complete the 10 MWT [21,30,34,36] and three studies recorded walking speed and distance throughout the 10 MWT [31,38,41]. A total of three studies assessed the 10 MWT at pre-intervention with powered exoskeleton assistance. Bach Baunsgaard et al. examined the 10 MWT within recently (35.3 s; range 26.5–44.1 s) and chronically (33.8 s; range 20.8–46.8 s) injured groups [30]. Jang et al. recorded the time required to walk 10 m at both a self-perceived comfortable (89.00 s) and self-perceived fast (76.42 s) walking speed [36]. Kressler et al. recorded 10 MWT walking speed and distance which ranged from 0.02 to 0.15 m·s^−1^ and 5 to 15 m, respectively [41]. The 10 MWT was recorded at mid intervention in four studies. Similar results were produced by both the recently (35.8 s; range 27.1–44.4 s) and chronically (33.2 s; range 20.2–46.2 s) injured groups [30]. Mixed results were recorded by Jang et al. with improvements made at a self-perceived comfortable (86.50 s) walking speed but no time improvements at a self-perceived fast (80.12 s) walking speed [36]. Benson et al. also recorded the 10 MWT at mid intervention (22–42 s) [31]. Walking speed and distance improved slightly from pre-to-mid (0.08–0.2 m·s^−1^; 8–20 m) intervention [41]. Post-intervention 10 MWT was recorded in five studies. Improvements were displayed in both the recently (28.6 s; range 20.0–37.1 s) and chronically (27.3 s; range 14.0–40.0 s) injured groups [30]. Jang et al. reported improvements when walking at both a self-perceived comfortable (86.47 s) and self-perceived fast (76.52 s) speed [36]. The results gathered by Benson et al. reduced in range (22 – 30 s) [31]. An increase in walking speed and distance (0.1–0.35 m·s^−1^, 10–35 m) was also reported [41]. Khan et al. recorded 10 MWT walking speed at post-intervention (0.43 ± 0.11 m·s^−1^) [38]. Benson et al. also recorded the 10 MWT without exoskeleton assistance at pre (24–341 s) and post (23–226 s) intervention among SCI patients [31]. Mean 10 MWT results recorded by Escalona et al. (57 s; range 38–89 s) and Farris et al. (58 ± 3.1 s) with exoskeleton assistance were considerably lower in comparison to unpowered exoskeleton assistance (96 ± 5.3 s) [21,34].

#### 3.4.3. TUG

A total of five studies performed the TUG test to assess gait performance. TUG was assessed at pre-intervention in three studies. The mean pre-intervention TUG results across the three studies were 33.4 ± 12.2 s, 35.0 s, 38.3 s and 76.16 s, respectively [28,30,36]. TUG was recorded at mid intervention in two studies [30,31]. The mean TUG time recorded by Benson et al. was 58.2 s and ranged from 50 to 74 s [31]. Bach Baunsgaard et al. showed an improvement at from pre-to-mid intervention in both chronically (31.4 s; range 17.4–45.4 s, CI = 95%) and recently (36.6 s; range 29.0–44.2 s, CI = 95%) injured groups [30]. A further improvement was displayed for TUG results at post-intervention by three authors (28.6 ± 13.7 s, 27.2 s, 31.3 s, 48.8 s) in their respective studies [28,30,31]. Jang et al. did not show any improvement at post-intervention with a TUG result of 76.68 s, with a single patient [36]. Farris et al. examined TUG in a single session with a powered exoskeleton (96 ± 6.6 s), which was completed faster when compared to unpowered exoskeleton assistance (111 ± 5.8 s) [34].

#### 3.4.4. 25 FWT and 2 MWT

Afzal et al. recorded walking speed during the 25FWT at both a self-selected walking speed (25FWT-SS) and fast walking speed (25FWT-FS) [28]. A slight improvement was made from pre-to-post-intervention in both the 25FWT-SS (0.35 ± 0.2 to 0.42 ± 0.2 m·s^−1^) and 25FWT-FS (0.51 ± 0.2 to 0.58 ± 0.3 m·s^−1^) [28]. Kozlowski et al. recorded the participants best performances during the 2 MWT throughout a 24-week intervention, total distance walked ranged from 13.8 to 24.9 m and walking speed ranged from 0.11 to 0.21 m·s^−1^ [40]. 

#### 3.4.5. 30 MWT

Kwon et al. recorded walking distance during the 30 MWT with both the ReWalk powered exoskeleton and unpowered KAFO [43]. Due to the fact patients were walking at a higher speed with the KAFO (8.4 ± 4.2 m·min^−1^, 22.6 ± 7.6 steps·min^−1^) in comparison to the powered exoskeleton (6.6 ± 1.2 m·min^−1^, 21.4 ± 2.4 steps·min^−1^), slightly greater distance was covered during the 30 MWT using the KAFO (242.9 ± 119.8 m) when compared to the ReWalk (196.2 ± 35.7 m) [43].

#### 3.4.6. Speed, Distance and Steps

As illustrated below, *n* = 11 of the included studies recorded walking speed with powered exoskeleton assistance (Table 5). Speed was reported as metres per minute (m·min^−1^) in two studies; the mean walking speed in both studies was 21.18 ± 1.75 m·min^−1^ and 13.8 ± 6.0 m·min^−1^, respectively [29,45]. Arazpour et al. found that walking speed with powered exoskeleton assistance was significantly faster (*p* = 0.000) than walking with unpowered assistance (HKAFO: 13.84 ± 1.85 m·min^−1^; IRGO: 15.28 ± 2.02 m·min^−1^) [29]. Walking speed was recorded as centimetres per second (cm·s^−1^) by Jang et al. which increased from pre (6.8 cm·s^−1^) to post (10.3 cm·s^−1^) intervention [36]. A further eight studies recorded speed as m·s^−1^, the results collectively ranged from 0.02 to 1.5 m·s^−1^ [14,21,22,32,34,38,40,41]. Walking distance per session was recorded in five studies; across the five studies the total distance collectively ranged from 62.83 to 1000 m [22,29,40,41,45]. The total number of steps was noted in nine studies. Escalona et al. recorded the total number of steps throughout the intervention which ranged from 1075 to 21,246 total steps [21]. The number of steps per minute (steps·min^−1^) was recorded in two studies; Jang et al. showed an improvement from pre (16.4 steps·min^−1^) to post (24 steps·min^−1^) intervention [36], and Chang et al. recorded a mean of 104 ± 11 steps·min^−1^ [32]. The remaining six studies calculated the number of steps per session (steps·session^−1^), the combined results ranged from 59 to 2616 steps·session^−1^ [30,35,38,40,41,44]. Total walking time collectively ranged from seven minutes to two hours per session [14,22,29,30,32,33,35,37,38,40,41,44,45]. 

Therefore, powered exoskeleton assisted walking may be an effective method to help individuals with neurological impairments improve their cardiovascular fitness. The increase in oxygen consumption [14,21,22,32,33,35,42,43,45,47], elevation in HR [14,21,22,32,33,40,47] and self-perceived RPE results indicate that patients were able to reach at least a moderate-intensity level of exercise [14,21,30,39,40]. METs and PCI results highlighted how exoskeleton assisted walking enabled patients to exercise at a much greater intensity in comparison to wheelchair propulsion and non-exoskeleton assisted walking [32,38]. As well as improving cardiovascular fitness, patients also enhanced their gait performance with improvements in the 6 MWT [28,31,40], 10 MWT [30,31,36,41] and TUG [28,30,31] and increased walk time, distance and total steps [22,36,44].

## 4. Discussion

The purpose of this review was to systematically search and analyse existing publications that examined energy expenditure and gait performance during powered exoskeleton training. A total of *n* = 23 manuscripts, which included SCI, MS and stroke patients as well as able-bodied participants were reviewed. The energy expenditure variables examined in the selected studies included, $\dot{V}$O_2_, HR, RPE, METs, PCI, RER, energy expenditure and BP. The gait performance parameters measured in the selected studies included a variety of assessment protocols, such as, the 6 MWT, 10 MWT, TUG, 25 FWT, 2 MWT, 30 MWT, total steps, distance and walking speed. The primary aim of the current study was to systematically review current literature on the effects of exoskeleton assisted gait training on cardiovascular function. Cardiovascular disease is one of the leading causes of mortality among individuals who suffer from paraplegia [48]. Therefore, it is vital for patients with paraplegia to have access to support methods to help them achieve the physical activity guidelines for health recommended by the WHO [20]. In turn, this may help reduce the risk of developing cardiovascular disease and its comorbidities. 

### 4.1. Cardiovascular Function

The included studies displayed an increase in $\dot{V}$O_2_ from seated to standing, and a further increase from standing to walking with powered exoskeleton assistance. Mean $\dot{V}$O_2_ decreased slightly from pre-to-post-intervention across two studies [36,41]. $\dot{V}$O_2_ was also examined among able-bodied participants during non-exoskeleton assisted walking in two studies and results were compared to powered exoskeleton assisted walking. The results reported by Chang et al. highlighted that powered exoskeleton assisted walking produced double the $\dot{V}$O_2_ compared to non-exoskeleton assisted walking among able-bodied individuals [32]; whereas Maher et al. found that the $\dot{V}$O_2_ during exoskeleton assisted walking among SCI patients was slightly less than the $\dot{V}$O_2_ during non-exoskeleton assisted walking within an able-bodied control group [45]. Chang et al. used one group of able-bodied participants, who walked at a similar speed both with (1.2 ± 0.2 m·s^−1^) and without (1.3 ± 0.2 m·s^−1^) exoskeleton assistance [32], whereas Maher et al. used an experimental (SCI) and a control group [45]. The able-bodied control group used by Maher et al. walked almost six times faster (78.0 ± 10.5 m·min^−1^) than the SCI group (13.8 ± 6.0 m·min^−1^) [45]. When comparing both groups, the discrepancy in walking speed may be responsible for the lower $\dot{V}$O_2_ within the SCI group [45]. When walking without exoskeleton assistance, the able-bodied groups in both studies walked at a similar speed and generated similar $\dot{V}$O_2_ results, whereas when walking with exoskeleton assistance the group used by Chang et al. walked over five times faster and consumed over double the amount of oxygen than the SCI group in Maher et al. study [32,45]. Furthermore, Kwon et al. stated that walking with an unpowered KAFO elevated cardiorespiratory function to a greater level than powered exoskeleton walking, whereby patients were walking at a higher speed and cadence when using the KAFO, the non-standardisation in walking speed may be responsible for the discrepancy in cardiorespiratory recordings between devices [43]. A similar trend was present for HR data, which increased from seated to standing, and further increased from standing to walking with exoskeleton assistance. The results highlighted that powered exoskeleton assisted walking (57–177 bpm) produced a much higher HR [14,21,22,32,33,40,47], when compared to SCI patients using the lokomat and (84 ± 9 bpm) [33] and to non-exoskeleton walking among able-bodied participants (80–113 bpm) [32]. The RPE results indicated that exoskeleton assisted walking has the potential to produce a moderate-intensity level of exercise for individuals with neurological impairments. Knezevic et al. found a reduction in mean RPE from pre-to-post-intervention; this may indicate an improvement in cardiovascular fitness, as the participants found it easier to complete the same task [39]. 

Powered exoskeleton assisted walking produced higher METs (6.5 ± 1.0 METs) in comparison to non-exoskeleton walking (3.4 ± 0.6 METs) within an able-bodied population [32]. Jang et al. found a reduction of 1.2 METs from pre-to-post-intervention [36]. On average, able-bodied individuals walking at a speed of 1.3 m·s^−1^ produces 3.4 METs [49]; therefore, the results gathered by Chang et al. highlighted that walking with powered exoskeleton assistance produced almost double these METs, while walking at a slightly slower speed (1.2 ± 0.2 m·s^−1^) [32]. PCI during exoskeleton walking also presented a similar trend to the above variables. PCI during exoskeleton assisted walking was 4.3-fold greater than non-exoskeleton walking among able-bodied individuals [32] and, on average 3.34 ± 1.75 times higher than wheelchair propulsion [38]. Arazpour et al. and Farris et al. both highlighted that powered exoskeleton assisted walking produced a slightly lower PCI in comparison to walking with unpowered assistance (HKAFO and IRGO) [29,34]. Rampichini et al. stated that the net metabolic cost increased fourfold when walking with exoskeleton assistance at a self-selected comfortable speed in comparison to wheelchair propulsion at a self-selected comfortable speed (*p* < 0.001) [47]. 

The RER results collected suggested that CHO utilisation was significantly higher than fat oxidation during powered exoskeleton assisted walking. Energy expenditure increased from seated to standing and further increased from standing to walking in both SCI and able-bodied control groups [35,45]. Energy expenditure was slightly lower in all three elements among SCI patients with powered exoskeleton assistance in comparison to an able-bodied control group [45]. This may also be due the control group walking almost six times faster than the SCI group [45]. When walking with exoskeleton assistance, CHO utilisation was significantly higher than fat oxidation, which suggested that the use of a powered exoskeleton produced a higher exercise intensity, as CHO utilisation increases, and fat oxidation decreases with an increase in exercise intensity [50]. A reduction in energy expenditure variables from pre-to-post-intervention may be an indication that powered exoskeleton gait training enhanced cardiovascular function. Furthermore, the powered exoskeleton may enable patients to achieve the physical activity guidelines for health recommended by the WHO for SCI patients [20]; thus, reducing the risk of developing cardiovascular disease [15] and improving health [20].

### 4.2. Gait Performance 

As well as measuring an element of energy expenditure, some included studies (*n* = 21) also assessed gait performance parameters associated with powered exoskeleton assisted walking. Notable improvements were present from pre/mid-to-post-intervention across the 6 MWT [28,31,40], 10 MWT [30,31,36,41] and TUG [28,30,31]. Powered exoskeletons enabled participants to walk further during the 6 MWT, complete the 10 MWT 70% faster, and improved the time required to complete the TUG test by an average of 15 s, in comparison to an unpowered exoskeleton [34,37]. Improvements were also recorded in the 25FWT at both a self-selected speed and a fast speed from pre-to-post-intervention among patients with MS [28]. Jang et al. reported an improvement in both walking speed and distance from pre-to-post-intervention [36]. Lester and Gorgey, conducted two sessions with a powered exoskeleton and an increase in the number of steps was noted from session one to session two [44]. Evans et al. recorded improvements in walking speed and distance from walk one to walk two [22]. Therefore, as well as improving cardiovascular function, a powered exoskeleton walking intervention has the potential to enhance gait function among SCI, MS and stroke patients. However, Khan et al. conducted a follow up assessment two to three months post exoskeleton training, the results demonstrated that 10 MWT walking speed was slightly slower (0.01 ± 0.01 m·s^−1^) and less walking distance was covered during the 6 MWT (5.0 ± 1.4 m) [38], therefore further research may be necessary to explore the longitudinal effects of powered exoskeleton gait training.

The results demonstrated that powered exoskeleton assisted walking enabled participants to exercise at a moderate-intensity, mainly by elevating oxygen consumption and HR (Table 3). The elevation in oxygen consumption could potentially cause RMR to remain elevated for up to 24 h post-exercise [51]. This is mainly due to excess post-exercise oxygen consumption (EPOC), which can vary depending on exercise intensity and duration [51]. The EPOC effect is characterized by two phases; the first phase is the recovery of myocellular homeostasis immediately after exercise, and the second phase is the cellular contributions to exercise adaptations. The phases include the re-synthesis of adenosine triphosphate and phosphocreatine, the replenishment of oxygen stores, restoration of fluid and fuel stores and lactate oxidation and removal [51]. The EPOC effect can potentially increase energy expenditure in the hours post exercise, thus increasing the number of calories burned [51]. The enhancement of gait function resulted in patients being able to walk faster and further for a longer duration (Table 4), consequently increasing exercise intensity. The increase in exercise intensity may elevate EPOC and increase RMR to a higher rate for a longer duration post exercise. This in turn may support the findings of Karelis et al. and cause a reduction in body fat mass [23]. 

As well as improving cardiovascular function and gait performance, corresponding research has suggested that powered exoskeleton assisted training can potentially improve secondary health conditions [23,52,53,54,55,56]. Karelis et al. reported that exoskeleton gait training reduced body fat mass, increased lean body mass and improved bone mineral density [23]. Exoskeleton gait training has been shown to reduce pain after a single session [56] as well as post-intervention [52]. A significant reduction in spasticity was present, both after a single session [56] and post exoskeleton training intervention [54]. Participants reported self-perceived improvements in bladder, bowel [53,55] and cardiovascular function as well as a reduction in both pain and spasticity after an exoskeleton walking programme [53]. Improving walking ability may also have a positive effect on joint range of motion [57], in turn this may increase the patient’s ability to carry out daily tasks. Being more mobile and having the ability to independently carry out simple daily tasks could positively enhance the patient’s QoL [58]. 

Powered exoskeleton devices have been shown to be safe to use with patients who suffer from neurological impairments, with no falls, no serious adverse events [52], no fractures or major skin effects [59] or incidences of autonomic dysreflexia [60,61] reported by previous studies. Participant feedback after walking with exoskeleton assistance was very positive, with self-perceived improvements in physical strength, mood and mental state reported by SCI patients [55]. Powered exoskeleton training was also responsible for an improvement in mood and self-satisfaction, a reduction in stress levels [62], and an enhancement in both self-confidence and self-image among SCI patients [53]. The exoskeleton enabled patients to stand up right and talk to other people at eye level rather than looking up from a seated position, this led to an improvement in psychological and emotional state [63]. Being able to engage with others at eye level also had less strain on the patient’s neck [63]. As well as enhancing both physical and mental health, research suggested that patients felt that a powered exoskeleton device enabled them to focus on gait, balance and core stability more than any other form of assisted walking [64]. From a physiotherapists point of view, using a powered exoskeleton can strengthen rehabilitation practice in many ways, for example, increasing the work capacity of the patient and the therapist per session [64]. The exoskeleton prevented early onset of fatigue in both the therapist and the patient in comparison to unassisted over ground training, which enables the patient to walk further. Patients were able to remain upright for a longer duration before tiring and were able to safely rest in the device, which improved the quality and quantity of the sessions [64]. 

### 4.3. Limitations

**Figure 3:**
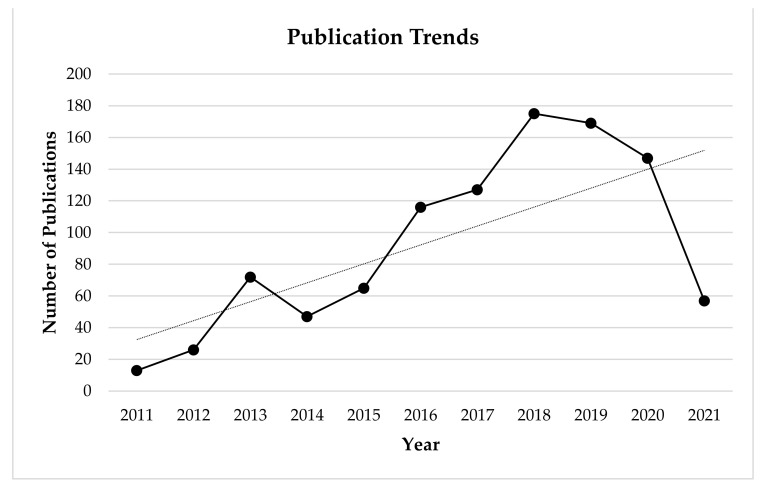
Google Scholar publication trends based on search term results over the past decade.

Study limitations include the sample sizes of some of the selected studies. Research in this area is sparse, albeit as illustrated below publications surrounding energy expenditure associated with powered exoskeletons has increased over the past decade (Figure 3). As a result some of the included studies were comprised of pilot studies and case studies/series which were made up of limited sample sizes. For example, the included pilot studies and case studies/series (*n* = 8) were all made up sample sizes ranging from (1–10) participants. Although the findings reported by the studies which consisted of smaller sample sizes (*n* = 1–10) were consistent with the results of studies made up of larger sample sizes (*n* > 12); having a larger sample size may allow results to be generalised to a larger population. It may be suggested that funding is another major restriction for therapists as a powered exoskeleton device is expensive therapeutic tool. 

### 4.4. Future Scope

Further research is necessary with larger sample sizes in order to support the findings of current literature. Future research should include control groups for further comparison of results. Further research is also necessary to explore the longitudinal effects of exoskeleton gait training on cardiovascular function, walking ability and secondary health conditions as the longest study duration was 24 weeks. 

## 5. Conclusions

In conclusion, the studies included within this systematic review examined the effects of exoskeleton gait training on cardiovascular function and gait performance. The included studies indicated that powered exoskeleton assisted training may increase oxygen consumption to a similar level to non-exoskeleton walking and elevate HR to a greater level than non-exoskeleton waking. The metabolic equivelant and the physiological cost index of exoskeleton assisted walking were shown to be three to four times greater than non-exoskeleton walking and wheelchair propulsion. Respiratory exchange ratio results highlighted that carbohydrate utilisation was higher during exoskeleton walking in comparison to non-exoskeleton walking. Energy expenditure was slightly less than non-exoskeleton walking and rate of percieved exertion reported during exoskeleton assisted walking was equal to moderate-intensity. The studies demonstrated an improvement in gait performance parameters from pre-to-post-interventions with patients improving across the six-minute walk test, ten-metre walk test, time up and go test and 25 foot walk test, as well as increasing walking speed, distance and total number of steps. 

Therefore, powered exoskeleton gait training may be an effective way to improve cardiovascular function and walking ability within spinal cord injury, multiple sclerosis and stroke patients. This enabled patients to walk faster and further for a longer duration, and as a result may enhance quality of life. An exoskeleton training programme has been shown to diminish secondary health conditions such as, pain and spasticity, improving bowel and bladder function, increasing bone mineral density [23,52,53,54,55,56]; as well as reducing stress, enhancing psychological state, mood and confidence [53,55]. A powered exoskeleton device is safe to use and does not cause any harm to the patient or therapist, the device can have a positive role to play in a rehabilitation programme by improving the quality and quantity of the sessions. The powered exoskeleton appears to be a novel method of facilitating paraplegic individuals to achieve physical activity guidelines for health recommended by the World Health Organisation [20]. This may decrease their risk of developing cardiovascular diseases, improve their gait and as a result positively impact their physical and mental health leading to an improved quality of life for the individual.

## Figures and Tables

**Table 1:** Study characteristics.

Authors	Sample Size (N)	Age (Years)	M/F	Injury	Device	Duration	Total Sessions	Sessions per Week	Session Duration	EE Parameters	Gait Parameters
Afzal et al. [28]	10	54.3 ± 12.4	M2/F8	MS	EKSO	3 weeks	15	5	90 min	$\dot{V}$O_2_ peak	25F WT, 6 MWT, TUG
Arazpour et al. [29]	4	26.8 ± 2.94	M2/F2	SCI	PGO	8 weeks	18	3	2 h	PCI	Walking speed and distance
Asselin et al. [14]	8	24–61	M7/F1	SCI	ReWalk	1 testing session	1	-	-	$\dot{V}$O_2_, HR, RPE	Sit, stand and walk speed
Bach Baunsgaard et al. [30]	52	35.8	M36/F16	SCI	EKSO and EKSO GT	8 weeks	24	3	-	HR, RPE	10 MWT, TUG, steps
Benson et al. [31]	10	23–43	M10	SCI	ReWalk	10 weeks	20	2	-	HR	10 MWT, 6 MWT, TUG
Chang et al. [32]	6	27–66	M4/F2	Able-Bodied	Hybrid neuro-prosthesis Exoskeleton	4 weeks	-	-	-	$\dot{V}$O_2_, HR, PCI, METS	Walking speed, steps
Corbianco et al. [33]	15	40 ± 15	M10/F5	SCI	Ekso GT	17 sessions	17	2	60 min	$\dot{V}$O_2_, METS, HR	EE recorded over a 10-min walk
Escalona et al. [21]	13	26.7–63.1	M8/F5	SCI	Ekso GT	6 weeks	18	2–3	-	$\dot{V}$O_2_, RER, HR, RPE	1 min sit, 1 min stand, 10 MWT, speed
Evans et al. [22]	5	28–51	M4/F1	SCI	Indego	2 testing sessions	2	-	-	$\dot{V}$O_2_, HR, METs	6 MWT, speed
Farris et al. [34]	1	42	M1	SCI	Vanderbilt (Indego)	1 testing session	1	1	-	PCI	TUG, 10 MWT, 6 MWT
Gorgey et al. [35]	4	21–57	M4	SCI	EKSO	15 weeks	15	1	60 min	$\dot{V}$O_2_ (L·min^−1^), energy expenditure	Walk time, steps
Jang et al. [36]	1	57	M1	SCI	Angelegs	6 weeks	30	5	30 min	METs, $\dot{V}$O_2_ peak	10 MWT, TUG, speed and steps
Jayaraman et al. [37]	12	57.8 ± 7.2	M8/F4	Stroke	SMA	1 testing session	1	-	30–45 min	$\dot{V}$O_2_	6 MWT
Khan et al. [38]	12	37.5 ± 13.7	M8/F4	SCI	ReWalk	12 weeks	>40	3.7 ± 0.2	1 h	PCI	10 MWT, 6 MWT, TUG, speed and steps
Knezevic et al. [39]	5	18–65	M5	SCI	ReWalk	60 sessions	60	-	-	$\dot{V}$O_2_, RPE	6 MWT
Kozlowski et al. [40]	7	21–49	M7	SCI	EKSO	24 weeks	24	1	2 h	HR, METs, RPE	Walk time, distance, steps and 2 MWT
Kressler et al. [41]	3	26–38	M2/F1	SCI	EKSO	6 weeks	18	3	60 min	$\dot{V}$O_2_ peak, HR, substrate utilisation	Walking Speed, 2 MWT
Kressler et al. [42]	4	24–48	M1/F3	SCI	Ekso GT	2 testing sessions	2	-	-	$\dot{V}$O_2_, energy expenditure, substrate utilisation	3 × 6 min walking bouts
Kwon et al. [43]	10	31 ± 10.3	M8/F2	SCI	ReWalk	2 × 4-week blocks	40	-	60–90 min	$\dot{V}$O_2_, HR, METs, PCI, energy expenditure	6 MWT, 30 MWT
Lester and Gorgey, [44]	1	21	M1	SCI	EKSO	3 weeks	3	1	Based on walk time	HR, BP	Steps
Maher et al. [45]	20	18–60	-	SCI (10)	Ekso GT	2–4 weeks	4	1	45 min	$\dot{V}$O_2_, RER, energy expenditure,	Distance and Speed
Postol et al. [46]	32	56.5 ± 11.4	M22/F10	Stroke (6), MS (6), Healthy (20)	REX Bionics	12 weeks	24	2	-	$\dot{V}$O_2_	5-min walk time
Rampichini et al. [47]	1	28	F1	SCI	ReWalk	1 testing session	1	-	-	$\dot{V}$O_2_, HR	Speed

**Table 2:** Mean $\dot{V}$O_2_ results (mL·kg^−1^·min^−1^, mL·kg^−1^ km^−1^, L·min^−1^ or mL) during exoskeleton assisted ambulation.

Author	Exoskeleton Ambulation
Afzal et al. [28]	Pre 6 MWT: 5.76 ± 1.3 (N$\dot{V}$O_2_); Post 6 MWT: 5.91 ± 1.2 (N$\dot{V}$O_2_) Pre 25FWT-SS: 2.83 ± 1.1 (N$\dot{V}$O_2_); Post 25FWT-SS: 2.01± 1.0 (N$\dot{V}$O_2_) Pre 25FWT-FS: 3.60 ± 1.3 (N$\dot{V}$O_2_); Post 25FWT-FS: 3.31 ± 1.9 (N$\dot{V}$O_2_)
Asselin et al. [14]	Sit: 3.5 ± 0.4 mL·kg^−1^·min^−1^; Stand: 4.3 ± 0.9 mL·kg^−1^·min^−1^; Walk: 11.2 ± 1.7 mL·kg^−1^·min^−1^
Chang et al. [32]	Exo: 22.5 ± 3.4 mL·kg^−1^·min^−1^ Non Exo: 11.7 ± 2.0 mL·kg^−1^·min^−1^
Corbianco et al. [33]	Sit: 2.58 ± 0.67 mL·kg^−1^·min^−1^; Stand: 3.02 ± 0.48 mL·kg^−1^·min^−1^; Walk: 7.73 ± 1.02 mL·kg^−1^·min^−1^
Escalona et al. [21]	Sit: 2.7–3.1 mL·kg^−1^·min^−1^; Stand: 3.8–5.3 mL·kg^−1^·min^−1^; Walk: 5.9–7.8 mL·kg^−1^·min^−1^
Evans et al. [22]	Walk 1: 9.5 ± 0.8 mL·kg^−1^·min^−1^; Walk 2: 11.5 ± 1.4 mL·kg^−1^·min^−1^
Gorgey et al. [35]	Sit: 0.27 L·min^−1^; Stand: 0.4 L·min^−1^; Walk: 0.55 L·min^−1^
Jang et al. [36]	Pre: 1208.1 mL; Mid: 1077.9 mL; Post: 901.3 mL
Jayaraman et al. [37]	34.92 ± 14.84 mL·kg^1^·km^−1^(6 MWT) and 0.08 ± 0.04 mL·kg^−1^·km^−1^ (graded treadmill test) improvement with a powered exoskeleton.
Knezevic et al. [39]	Pre: 9.76 ± 1.23 mL·kg^−1^·min^−1^; Mid: 10.93 ± 1.90 mL·kg^−1^·min^−1^; Post: 12.73 ± 2.30 mL·kg^−1^·min^−1^
Kressler et al. [41]	Pre: 21.6 mL·kg^−1^·min^−1^; Post: 20.8 mL·kg^−1^·min^−1^
Kressler et al. [42]	16.6 mL·kg^−1^·min^−1^ (7.7–25.3 mL·kg^−1^·min^−1^) $\dot{V}$O_2_ peak
Kwon et al. [43]	Mean: 6 MWT: 9.0 ± 2.1 mL·kg^−1^·min^−1^; 30 MWT: 8.8 ± 1.8 mL·kg^−1^·min^−1^ Peak: 6 MWT: 19.3 ± 6.8 mL·kg^−1^·min^−1^; 30 MWT: 23.2 ± 7.4 mL·kg^−1^·min^−1^
Maher et al. [45]	Exo: Sit: 4.3 ± 1.12 mL·kg^−1^·min^−1^; Stand: 4.7 ± 0.58 mL·kg^−1^·min^−1^; Walk: 8.5 ± 0.90 mL·kg^−1^·min^−1^ Non-Exo: Sit: 4.2 ± 0.44 mL·kg^−1^·min^−1^; Stand: 4.8 ± 0.45 mL·kg^−1^·min^−1^; Walk: 11.3 ± 1.30 mL·kg^−1^·min^−1^
Postol et al. [46]	Exo: Pre: 3.7 ± 0.7 mL·kg^−1^·min^−1^; Post: 5.3 ± 1.9 mL·kg^−1^·min^−1^ Non-Exo: 8.6 ± 1.4 mL·kg^−1^·min^−1^
Rampichini et al. [47]	12.4–15.5 mL·kg^−1^·min^−1^

**Table 3:** Mean HR (bpm) and RPE (Borg scale) data of exoskeleton assisted ambulation.

Authors	Sit/Rest (bpm)	Stand (bpm)	Exoskeleton Walking (bpm)	Post Walk (bpm)	RPE (Borg Scale)
Asselin et al. [14]	70 ± 10	81 ± 12	118 ± 21	-	10 ± 2 (7–13)
Bach Baunsgaard et al. [30]	-	-	15–21% increase in HR from sitting to walking	-	13 (11–13) over 24 sessions
Benson et al. [31]	82.6	-	-	91.4	-
Chang et al. [32]	-	-	Exo: 148 ± 19 Non-Exo: 99 ± 14	-	-
Corbianco et al. [33]	76 ± 12	85 ± 9	100 ± 13	-	-
Escalona et al. [21]	74	89	114	-	3.2
Evans et al. [22]	-	-	Walk 1: 121 ± 30 Walk 2: 142 ± 35	-	-
Knezevic et al. [39]	-	-	-	-	Reduced from 13 ± 5.95 to 7 ± 3.52
Kozlowski et al. [40]	69.5 (52–115)	-	103.7 (57–136)	89 (53–135)	9.7 (6–20)
Kressler et al. [41]	-	-	Pre: 166.6 ± 24.0 (139–182) Post: 172.6 ± 5.13 (167–177)	-	-
Kwon et al. [43]	-	-	6 MWT; mean: 112.5 ± 13.6 peak: 124.8 ± 16.1 30 MWT; mean: 118.6 ± 14.6, peak: 131.9 ± 19.3	-	-
Lester and Gorgey, [44]	Session 1: 69 Session 2: 82	-	-	Session 1: 74 Session 2: 60	-
Rampichini et al. [47]	87	94	115	-	-

**Table 4:** Mean 6 MWT (m) and 10 MWT (s or m·s^−1^) results.

Author	6 MWT	10 MWT
Afzal et al. [28]	Pre: 121.2 ± 64.7 m Post: 128.0 ± 63.4 m	-
Bach Baunsgaard et al. [30]	-	Pre: 35.3 and 33.8 s Mid: 35.8 and 33.2 s Post: 28.6 and 27.3 s
Benson et al. [31]	Mid: 117.6 m (50–162 m) Post: 140.8 m (91–174 m)	Mid: 30 s Post: 24.4 s
Escalona et al. [21]	-	57 (38–89) s
Evans et al. [22]	Walk 1: 67.4 ± 3.8 m, Walk 2: 95.9 ± 18.6 m	-
Farris et al. [34]	64 ± 4.5 m	58 ± 3.1 s
Jang et al. [36]	-	Pre: 89 and 76.42 s Mid: 86.5 and 80.12 s Post: 86.4 and 76.52 s
Jayaraman et al. [37]	32.3 ± 15.5 m improvement with powered vs. unpowered exoskeleton assistance.	-
Khan et al. [38]	Post: 146.3 ± 35.3 m	Mean speed of 0.43 ± 0.11 m·s^−1^ post-intervention
Knezevic et al. [39]	Pre: 51.1 ± 51 m Mid: 71.9 ± 45.1 m Post: 98.9 ± 42.3 m	-
Kressler et al. [41]	-	Pre: 0.02–0.15 m·s^−1^ Mid: 0.08–0.2 m·s^−1^ Post: 0.1–0.35 m·s^−1^
Kwon et al. [43]	Powered: 39.1 ± 5.4 m Unpowered: 50.9 ± 25.5 m	-

**Table 5:** Mean walking speed (m·min^−1^, cm·s^−1^ or m·s^−1^), distance (m) and steps (steps·min^−1^ or total steps).

Author	Speed (m·min^−1^, cm·s^−1^ or m·s^−1^)	Distance (m)	Steps (Steps·min^−1^ or Total Steps)
Arazpour et al. [29]	21.18 ± 1.17 m·min^−1^	120 ± 12.98 m	-
Asselin et al. [14]	0.22 ± 0.11 m·s^−1^	-	-
Bach Baunsgaard et al. [30]	-	-	350–1200 steps·session^−1^
Chang et al. [32]	1.2 ± 0.2 m·s^−1^	-	104 ± 11 steps·min^−1^
Escalona et al. [21]	0.18 m·s^−1^ (0.11–0.26 m·s^−1^)	-	1075–21,246 steps
Evans et al. [22]	1: 0.19 ± 0.01 m·s^−1^ 2: 0.27 ± 0.05 m·s^−1^	1: 67.40 ± 3.76 m 2: 95.93 ± 18.64 m	-
Farris et al. [34]	0.063–0.18 m·s^−1^	-	-
Gorgey et al. [35]	-	-	59–2284 steps·session^−1^
Jang et al. [36]	Pre: 6.8 cm·s^−1^ Mid: 9.7 cm·s^−1^ Post: 10.3 cm·s^−1^	-	Pre: 16.4 steps·min^−1^ Mid: 23.9 steps·min^−1^ Post: 24 steps·min^−1^
Khan et al. [38]	0.43 ± 0.11 m·s^−1^ (0.28–0.60 m·s^−1^)	-	1359 ± 692 steps·session^−1^
Kozlowski et al. [40]	0.15 m·s^−1^ (0.11–0.21 m·s^−1^)	385.3 m (110–670 m)	1525 steps·session^−1^ (561–2616 steps·session^−1^)
Kressler et al. [41]	0.02–0.35 m·s^−1^	50–1000 m	200–2600 steps·session^−1^
Lester and Gorgey, [44]	-	-	Session 1: 83 steps Session 2: 589 steps
Maher et al. [45]	13.8 ± 6.0 m·min^−1^	604.3 ± 278.4 m	-

## Data Availability

Not applicable.

## Abbreviations

**Abbreviation**	**Explanation**
$\dot{V}$O_2_	Volume of oxygen
10 MWT	Ten-metre walk test
25 FWT	25-foot walk test
2 MWT	Two-minute walk test
30 MWT	30-min walk test
6 MWT	Six-minute walk test
BP	Blood pressure
bpm	Beats per minute
CHO	Carbohydrate
cm·s^−1^	Centimetres per second
EE	Energy expenditure
EPOC	Excess post-exercise oxygen consumption
g·min^−1^	Grams per minute
HKAFO	Hip knee ankle foot orthosis
HR	Heart rate
HR_Res_	Heart rate reserve
IRGO	Isocentric reciprocating gait orthosis
KAFO	Knee ankle foot orthosis
kcal·min^−1^	Calories per minute
km·h^−1^	Kilometres per hour
L·min^−1^	Litres per minute
m	Metres
MET	Metabolic equivalent of task
mL	Millilitres
mL·kg^−1^·km^−1^	Millilitres per kilogram of body mass per kilometre
mL·kg^−1^·min^−1^	Millilitres per kilogram of body mass per minute
mmHg	Millimetres of mercury
m·min^−1^	Metres per minute
MS	Multiple sclerosis
m·s^−1^	Metres per second
PRISMA	Preferred reporting items for systematic reviews and meta-analyses
QoL	Quality of life
RER	Respiratory exchange ratio
RMR	Resting metabolic rate
RPE	Rate of perceived exertion
SCI	Spinal cord injury
s	Seconds
steps·min^−1^	Steps per minute
steps·session^−1^	Steps per session
TUG	Time up and go test
WHO	World Health Organisation

## Appendix A

Search terms and database results.

Databases searched—Google Scholar and Science Direct (2 April 2020 to 12 February 2021).

Search terms and results:

1. “Energy expenditure” of “powered exoskeleton” ambulation (Scholar 481, Science Direct 6).
2. “Cardiorespiratory demands” of “powered exoskeleton” walking (Scholar 2, Science Direct 0).
3. “Metabolic demands” of “powered exoskeleton” walking (Scholar 24, Science Direct 1).
4. Ekso GT energy cost (Scholar 140, Science Direct 3)
5. “Powered exoskeleton” “energy expenditure” paraplegic (Scholar 298, Science Direct 5).
6. “Powered exoskeleton” “physiological cost” paraplegic (Scholar 91, Science Direct 2).
7. “Powered exoskeleton” “oxygen demand” paraplegic (Scholar 130, Science Direct 0).
